# The Poor Man's Home.—XIX

**Published:** 1897-12-25

**Authors:** 


### Modern Sociology.

### THE POOR MAN'S HOME.—XIX.

### Housing in Germany.

Germany has largely imitated England in its experiments in housing. Many societies avow that they are following in the footsteps of Miss Octavia Hill, and it seems to be from Britain that the whole impetus to reform in this matter has come. Nevertheless, there is sufficient difference between the German and English ideals to make it worth while to give some account of how they manage these things in the Fatherland.

In Berlin the Mutual Building Society has erected a block of tenements for working people which are very like those to be found in London. The chief difference is that the court behind the building is partitioned off so that each section of the block has its own court separate from its neighbours. This dividing of the yard or garden into more or less private portions is common in German tenements. Where, as in some places, it results in each family having a little garden of their own, we can see an advantage in it; but where this is impossible there seems to be little reason for it, though it may to a certain extent prevent that quarrelling over the right to drying-space which is sufficiently common among the house-wives who occupy tenements. The society we are speaking of is the owner of 50 buildings, containing in all 491 tenements. Of these 219 are of two rooms, 234 of three, and 38 of four. Those families who occupy the larger tenements have the advantage of having their sanitary conveniences for their private use, while in the two-room houses one closet only is provided for two families. In both cases these are placed on the staircase landing, are well-flushed, and are ventilated from the open air. In the cellar the laundries are placed, and each family has the use of one for two days in the month. Each kitchen has a large range, with an oven; in the other rooms heating is provided by porcelain stoves. An average two-room flat has a kitchen 13 ft. long by 9 ft. 4 in. wide, and a bedroom which measures 17 ft. or 17½ ft. by 13 ft. Three-room tenements contain, in addition, a very long narrow bedroom, 18 ft. long by 8½ ft. Ceilings throughout are 9 ft. 4 in. high.

The rent of the smaller flats vary from 150 to 205 marks per annum (£7 8s. 9d. to £10 3s. 3½d.). The rent of a three-room tenement runs from 300 to 315 marks (£14 17s. 6d. to £15 12s. 4½d.). The company was formed on a philanthropic basis, and dividends are limited to 4 per cent. Anything earned beyond this is employed in furthering the work. The fixed percentage has always been earned, and indeed the society seems to be very fortunate in its tenants. The average duration of tenancy, we are told, is 20 years. The rents absorb about 20 per cent. of the earnings of the heads of families who live in the tenements, but even at that they are nearly 25 per cent. cheaper than similar accommodation can be got for in the neighbourhood. The tenants, though respectable, are generally very poor. The highest income among them is 1,500 marks (about £74 7s. 6d.), and there are some widows and single women whose average earnings are not more than £22. Porters and janitors are chosen from among the oldest tenants. They receive no salary for the duties they fulfil, nor is any reduction made in their rents; their only emolument is a gratuity at Christmas amounting to 30 marks, or about 25s.

There has been more done in the way of providing workmen's dwellings in Leipsic than in Berlin. Leipsic has increased largely during the last twenty-five years, owing to the great development of industry in it; and, with the increase of population, house-room had grown dearer. Recognising that the proportion of their earnings which the working-classes had to spend on housing was unduly high, Mr. Herrman J. Meyer determined to build some model dwellings after the model of the Peabody Trust. He fixed the rents to give him 3 per cent. net on his investment, and has spent this net income in extending his work. The plan of the buildings is on a generous scale. Only about 28 per cent. of the ground has been built on. The remaining space forms a court in the centre of the block of buildings, the greater part of which is divided off into small gardens, which the tenants can have for a rent of a little under 8s. a year. Each of these gardens is fenced and locked, and the tenants have nearly all planted flowers in their plots, and some have erected summer-houses. Besides these private gardens, there is a drying-ground and a playground, which are common property.

The buildings are four storeys high, and are solidly built of brick. The cellar is paved with bricks, and each family has a space partitioned off for its own use. The laundry also occupies part of the cellar. There is no water led into the houses, though wells are provided in the court. The supply is unlimited, but so much is not likely to be used as if it could be obtained without the trouble of carrying it up. Another disadvantage of this absence of water in the dwellings is that the privies are dry, and a disinfectant is used in place of water. The refuse is taken away once a month. These privies are, however, as well situated as may be, in an extension of the staircase, entirely outside the dwellings. There is one of these for each family. The tenements are of one, three, and four rooms; there are none of two rooms. The single rooms measure 14 ft. by 9 ft. 10 in. The rental of them varies according to the height, from 41.60 marks on the top storey to 62.40 marks on the second (£2 1s. 3d. to £3 1s. 10½d.). Three-room tenements run from 130 marks on the fourth floor to 160 marks on the second. (The second floor is considered the best, and flats on the first rent at 10 marks per annum less.) Reckoned in our money, these rentals are £6 8s. 11d. for the top storey and £7 18s. 8d. for the best. These tenements contain a small kitchen, 8 ft. 8 in. by 7 ft. 5 in.; a living-room, 14 ft. 1 in. by 12 ft. 8 in.; and a bedroom, 14 ft. 1 in. by 8 ft. 2 in. In the four-room tenements the living room and one bedroom are the same size as in the three-room ones; but the kitchen is a shade larger, measuring 8 ft. 8 in. by 8 ft. 4 in.; and there is also another bedroom 8 ft. 5 in. long, and 8 ft. 2 in. wide. The rent of these runs from 155 marks to 200 marks (£7 13s. 8½d. to £9 18s. 4d.). The ceilings throughout are 9 ft. 6 in. high. These houses of Herr Meyer's are conspicuously cheap, and, except for the want of water in the dwellings, seem to be in every way satisfactory.

